# Adaptations of the flat jack test for its application in cob walls

**DOI:** 10.1016/j.mex.2020.101003

**Published:** 2020-07-22

**Authors:** Alejandro Jiménez Rios, Dermot O'Dwyer

**Affiliations:** Department of Civil, Structural and Environmental Engineering Trinity College Dublin, College Green, D02 Dublin, Ireland

**Keywords:** Minor destructive tests (mdt), Heritage conservation, Earthen Buildings

## Abstract

The single and double flat jack tests have been successfully applied for the first time in cob walls. This technique has been used for over 40 years in the field of cultural heritage conservation to investigate the levels of stress in different masonry and earthen wall typologies and to determine their mechanical properties [1]. Two standards [2], [3], [4], [5], [6], [7] have been developed for the application of the technique in masonry walls and this paper presents the adaptation of those methods for its application in cob walls.

• The control points have been pinned to the material instead of glued to the surface which has facilitated its installation and speed the process.
• A timber frame has been fabricated, to which the cutting device has been supported and strapped, to facilitate the cutting of the slot thus obtaining a better-quality cut and a safety increase for the ring saw operator.
• The connection of the hydraulics system has been slightly modified by connecting the pressure transducer next to the flat jack's inlet, instead of just after the pump, thus avoiding the recording of pressure peaks while manually pumping the oil.

## Graphical abstract

**Figure:**
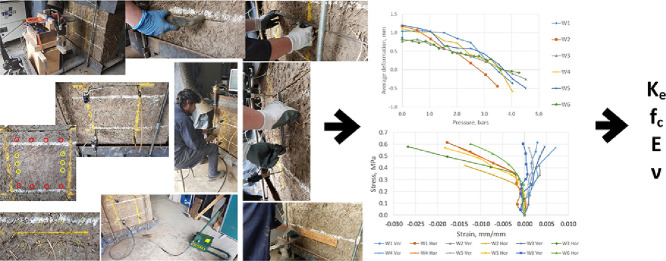

Specifications Table

**Table:** 

Co-submission paper:	Research article title: Experimental validation for the application of the flat jack test in cob wallsAuthors: Alejandro Jiménez Rios and Dermot O'DwyerJournal: Construction and Building Materials DOI: https://doi.org/10.1016/j.conbuildmat.2020.119148 Elsevier reference: JCBM 119,148 Article reference: JCBM_CONBUILDMAT-d-19–07,048
Subject Area	Engineering
More specific subject area:	Structural Engineering Minor Destructive Tests (MDT) Heritage Conservation Earthen Buildings
Method name:	Flat jack test for cob walls
Name and reference of original method	Flat jack test for masonry walls [2], [3], [4], [5], [6], [7]
Resource availability	This is paper is co-submitted with the main paper titled “Experimental validation for the application of the flat jack test in cob walls” [8] and correspondent co-submitted Data in Brief paper[9].

## Method details

Single flat jack tests were performed in six cob wallettes by performing the following steps:

1. Wallette setting up.
2. Control points fixing.
3. Initial distance between control points measured.
4. Slot cutting.
5. Cleaning of the slot.
6. Cut depth measurement.
7. Initial displacements measurement.
8. Hydraulic system connection and flat jack insertion.
9. System purging.
10. Seating pressure applied.
11. Removal of pressure.
12. Pressure increments.
13. Removal of pressure.
14. Removal of flat jack.

Double flat jack tests were performed in the same cob wallettes. The steps performed were as follows:

1. Wallette setting up.
2. Second slot cut.
3. Cleaning of the slot.
4. Second cut depth measurement.
5. Control points fixing.
6. Hydraulic system connection and flat jacks’ insertion.
7. System purging.
8. Seating pressure applied.
9. Removal of pressure.
10. Initial distance between control points measured.
11. Pressure increments.
12. Removal of pressure.

### Wallette setting up

**Fig. 1:**
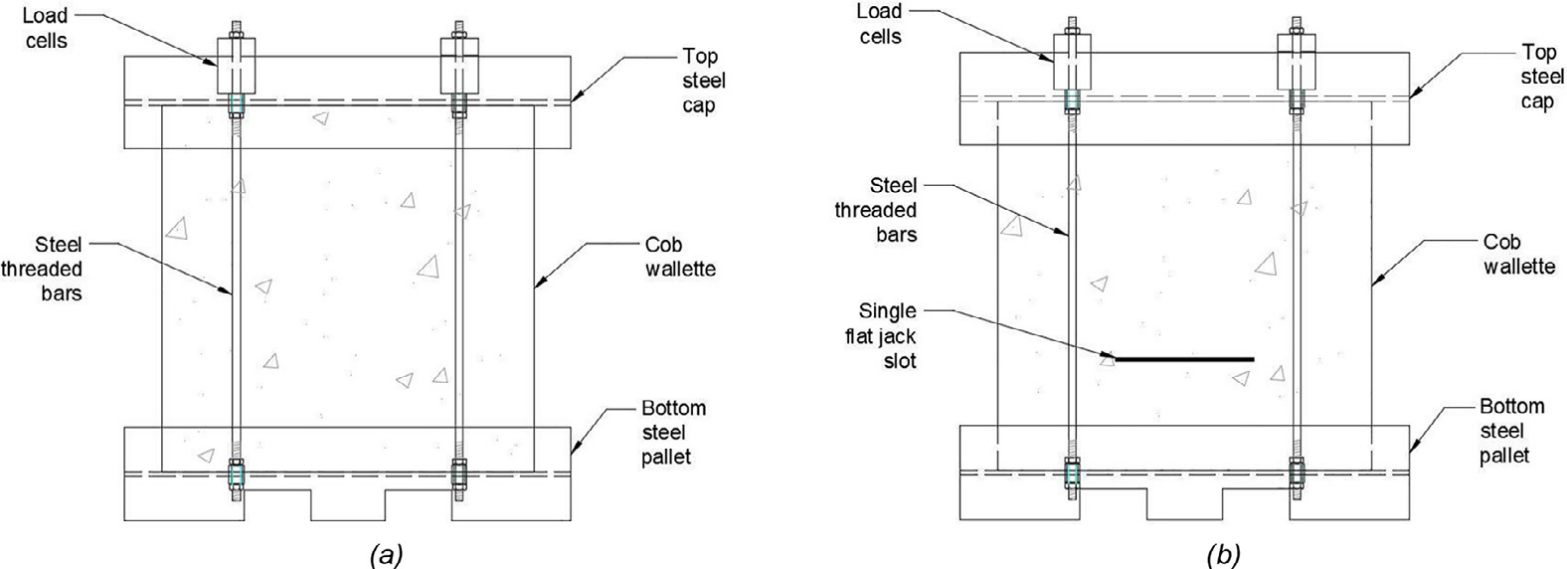
Cob wallette setup for (a) single flat jack test and (b) double flat jack test.

A load of 50 kN was imposed to the wallettes by manually tightening four steel threaded bars connected to a bottom steel pallet, upon which the wallettes were built, and a top steel cap. The tension forces thus applied caused the shortening of the bars, deformation that subsequently translated into a compressive force applied to the cob wallettes. Therefore, loads were applied slightly eccentrically but in a symmetric way (the points of anchorage of the bars were located at the external faces of the steel pallets flanges). The load applied to each bar was measured by a load cell placed between the nut and the point of support of the bar, which was provided by a piece of hollow steel welded to the flange of the top steel cap UB section. The load was applied in increments of approximately 2 kN per bar and the tightening order was alternated after each increment. The loading process took between 15 and 20 min for each one of the wallettes. The setup of the single flat jack tests is shown in Fig. 1(a).

The double flat jack test was performed in the same cob wallettes immediately after the single flat jack test had finished. Therefore, the setting up was similar. The compression forces acting in the wallettes, transferred by the tensed steel threaded bars, were kept in place. In this way, the top steel cap provided support to the material above the second cut and helped to prevent the failure of the material outside the slot area as highlighted in the standard [6]. The cob wallette setup for the double flat jack test is shown in Fig. 1(b) where the steel threaded bars and the slot created for the performance of the single flat jack test can be observed.

### Fixing of the control points

**Fig. 2:**
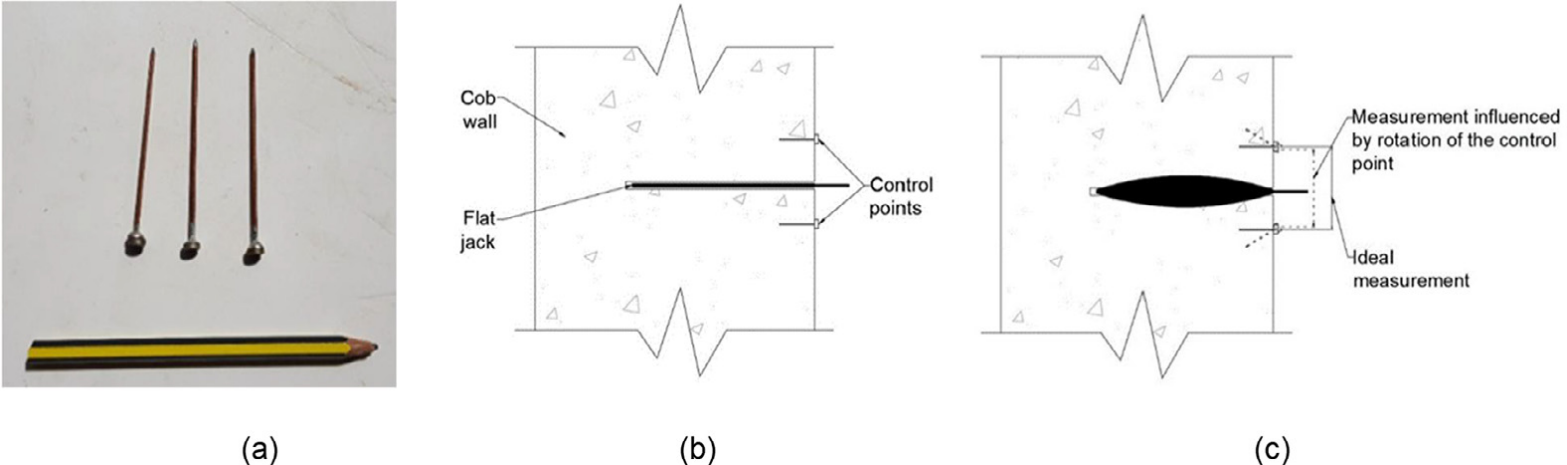
(a) Control points and sketch of nailed control points (a) before applying pressure in the flat jack and (b) after pressure is applied and the flat jack deforms.

Due to the nature of the cob wallettes’ surfaces, dusty and irregular, the use of a glue to fix the control points, as usually done in masonry walls, proved to be non-feasible. Therefore, it was decided to implement an alternative approach. Stainless steel control points were soldered to a 2 mm in diameter rod of copper and its tip was sharpened to create a kind of nail (see Fig. 2(a)). As cob is a relatively soft material this allowed us to nail the control points without causing much damage to the material. Moreover, this allowed to speed up the process as no time was wasted waiting for the glue to dry. The length of the rod proved to be an important factor to take into account though. If the rod was too short it would not be stable and the measurements could be completely invalidated. On the other hand, if the rod was too long it may rotate due to the non-uniform pressure transferred from the flat jacks to the material, as sketched in Fig. 2 (b and c), thus altering the measurements as well. After some trial and error attempts it was decided to use rod lengths of 4 cm as that length seemed to be the minimum length necessary to obtain a solid anchorage of the control point to the cob substrate.

**Fig. 3:**
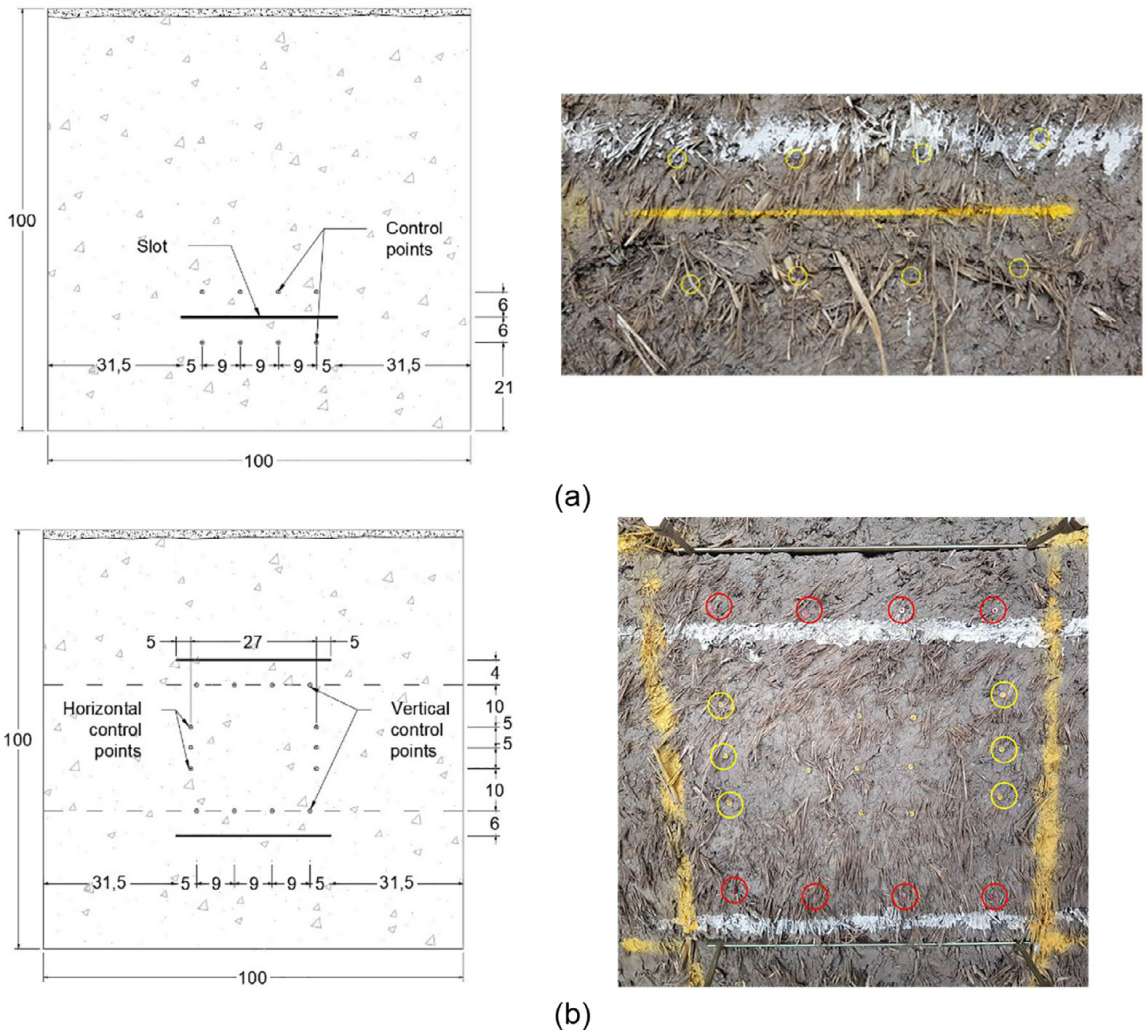
Sketch of control points' location (values in cm) and close up picture of installed control points in cob wallette for (a) single flat jack test and (b) double flat jack test.

The control points were approximately fixed for the single flat jack tests according to the distances shown in the sketch of Fig. 3(a) thus respecting the minimum and maximum ranges specified by the masonry standards [2], [3], [4]. Fig. 3(a) also shows the location of the cut and the 4 pairs of vertical control points fixed to one of the cob wallettes. The control points for the double flat jack tests were fixed approximately at the locations indicated in the sketch shown in Fig. 3(b). Four vertical points were nailed to the wallette's face taking as reference the four control points fixed above the lower slot for the application of the single flat jack test. Moreover, three horizontal control points were fixed at the middle third of the specimen's height. The horizontal control points were fixed at an initial distance of approximately 27 cm. With this arrangement it was possible to measure up to 3 cm of deformation as the maximum length capacity of the “Vernier” calipers used was 30 cm. Both vertical and horizontal control points fixed in one of the wallettes are shown in Fig. 3(b) where the vertical control points are highlighted with a red circle whereas that the horizontal control points are highlighted with a yellow circle.

### Measurement of control points’ initial distances

**Fig. 4:**
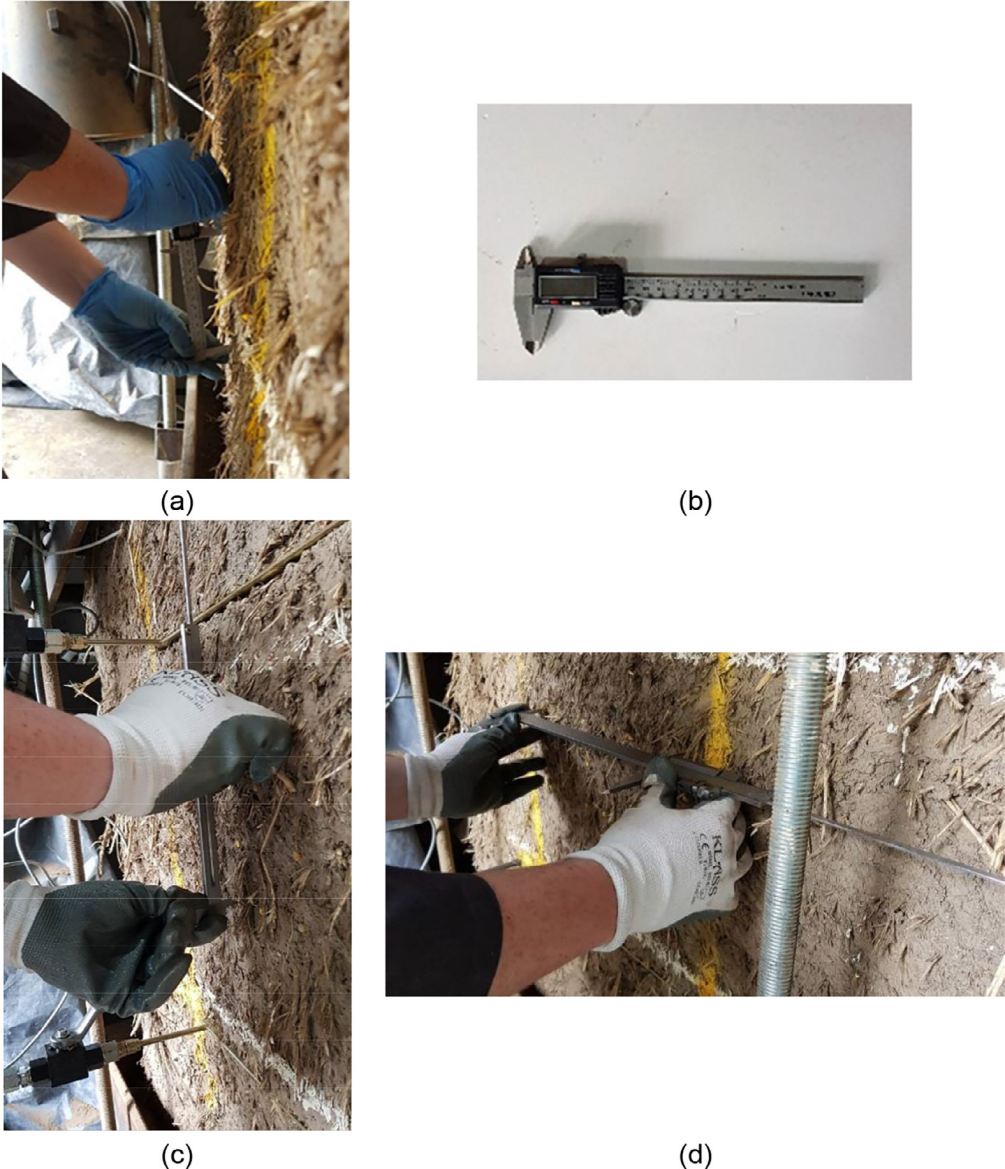
(a) Measurement of the initial distance between control points using a digital display caliper Vernier (b), measurement of the distances between (c) vertical control points and (d) horizontal control points for the double flat jack test.

Before proceeding with the cut of the slot for the single flat jack test, the initial distance between control points was measured (see Fig. 4(a)).The strain measuring equipment used was a “Vernier” calipers with a precision of 0.01 mm with a digital display (see Fig. 4(b)). The initial distance between control points for the double flat jack test was recorded after the purging of the system and its depressurization by using the “Vernier” calipers. Both distances between vertical and horizontal control points were respectively measured as can be seen in Fig. 4(c) and Fig. 4(d).

### Cutting

**Fig. 5:**
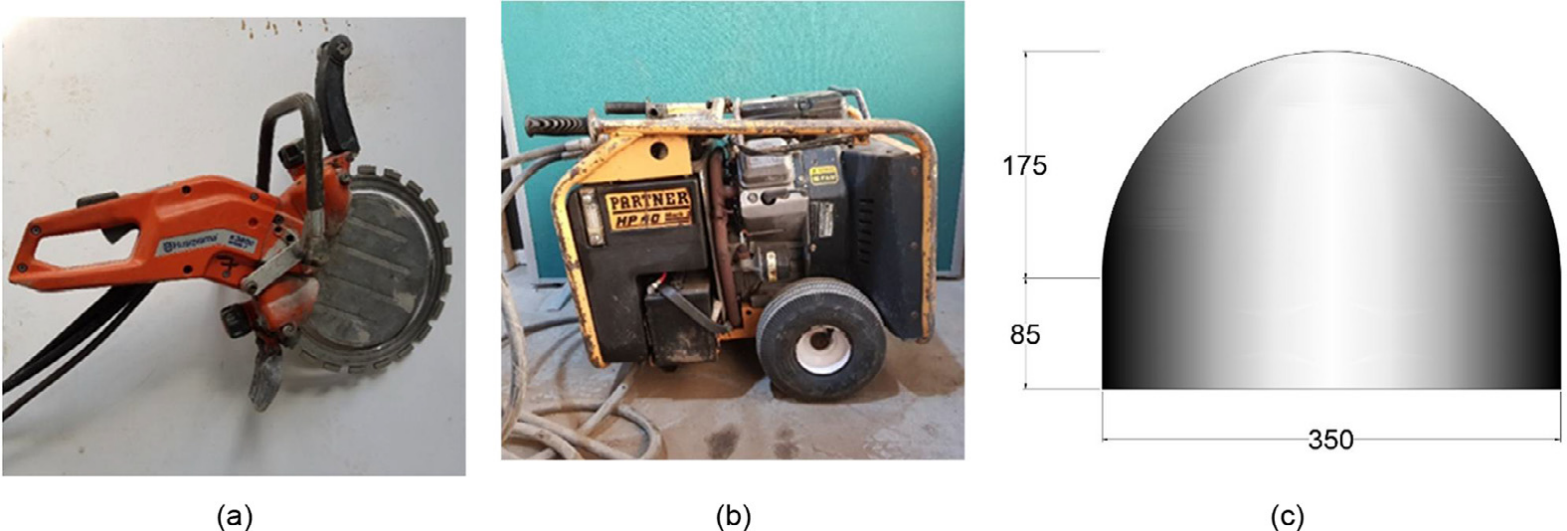
(a) Ring saw, (b) hydraulic power plant and eccentric flat jack dimensions (in mm).

The slot cutting equipment consisted in a ring saw model Husqvarna K3600 with a blade diameter of 370 mm and 6 mm thickness which allows for a maximum cut depth of 270 mm (see Fig.43 (a)). A bubble level was glued to the ring saw to serve as a guidance to the operator and ensure a horizontal cut. A power plant Husqvarna Power Pack PP518 was used to power the ring saw (see Fig. 43 (b)). The flat jacks used were eccentric flat jacks model SISGEO 0L103352600 with the dimensions shown in Fig. 43 (c) and a thickness of 4 mm Figs. 5,10.

**Fig. 6:**
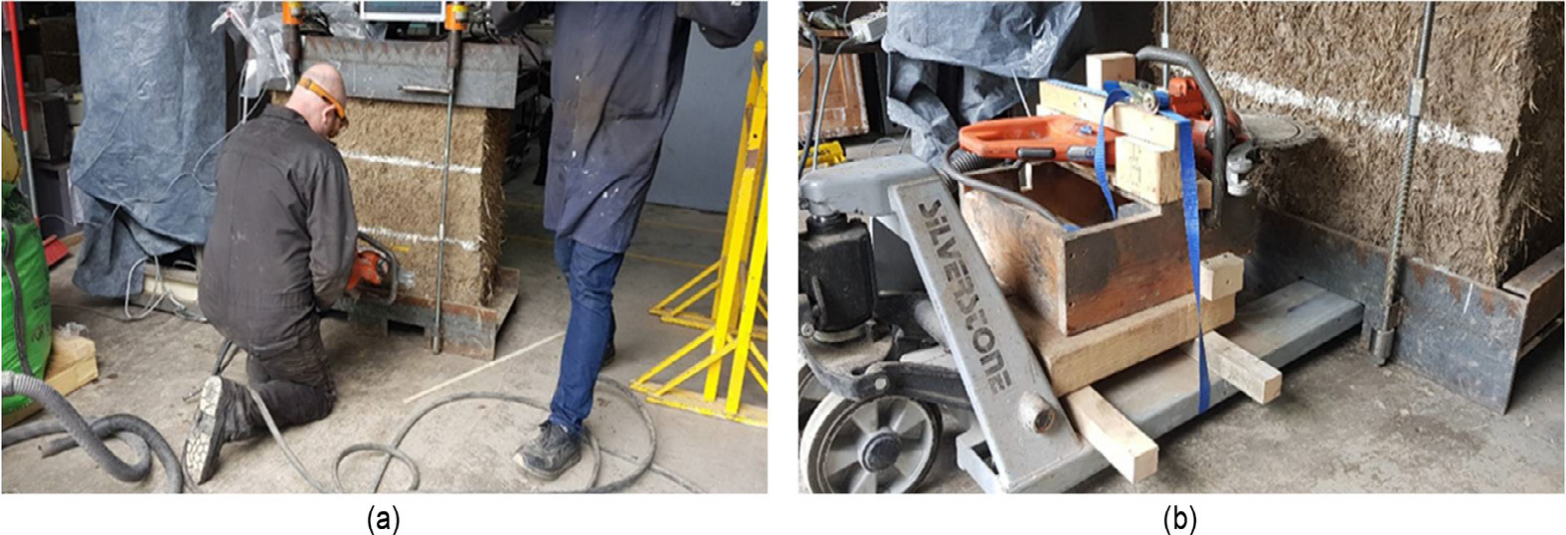
Cutting of the slot being performed (a) manually and (b) using the timber frame supported by the pallets jack.

To perform the cut in the first wallette (W6) the laboratory technician operated manually the ring saw. He had to kneel to make the cut at the required height (see Fig. 6(a)) and this position allowed him to use all his body as support to carry and stabilize the ring saw. During the execution of the cut he constantly monitored the bubble level to ensure horizontality. The result was a horizontal and good quality cut. Nevertheless, the technician expressed his discomfort, operating manually the ring saw in such an uncomfortable position, and his concern regarding the regularity and quality of future cuts.

**Fig. 7:**
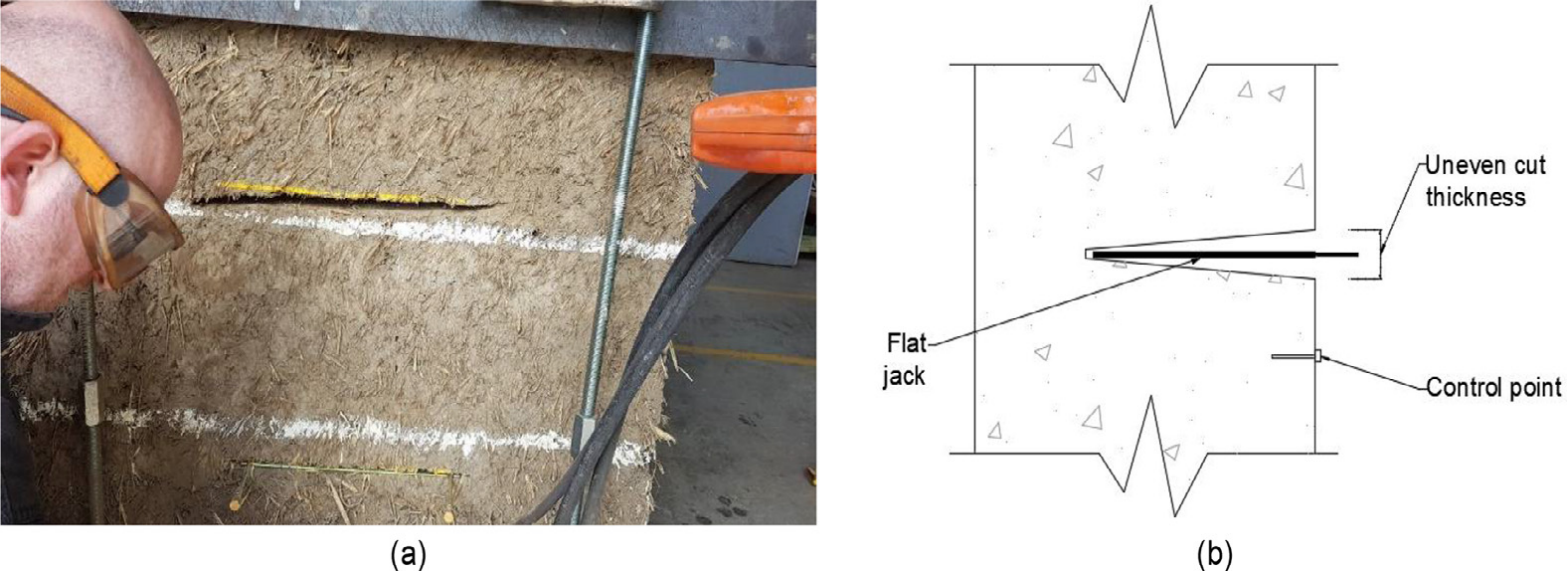
(a) Manual cut performed in the first cob wallette and (b) sketch showing the uneven cut thickness obtained.

The second cut in the first wallette was performed by the technician by manually operating the ring saw again. Unfortunately, as the cut had to be done at an awkward position and the technician had to carry all the weight of the ring saw in his arms, control the location of the cut and ensure its horizontality, all at the same time, the operation turned out to be too complicated and the quality of the cut was quite poor. As it can be observed in Fig. 7(a), the cut extended beyond the marked location, it was not horizontal and, as presented in the sketch of Fig. 7(b), the cut thickness was uneven, which prevented the use of steel shims to fill the space between the flat jack and the material.

Therefore, it was decided to fabricate a timber frame to which the ring saw could be attached and secured to facilitate the execution of the cut, obtain consistent results and avoid health and safety issues linked to the manual operation of the ring saw. The timber frame (see Fig. 6(b)) was supported over the pallets jack and the cut was simply performed by sliding the pallets jack under the bottom steel pallet. The implementation of the timber frame speeded up even further the application of the test.

**Fig. 8:**
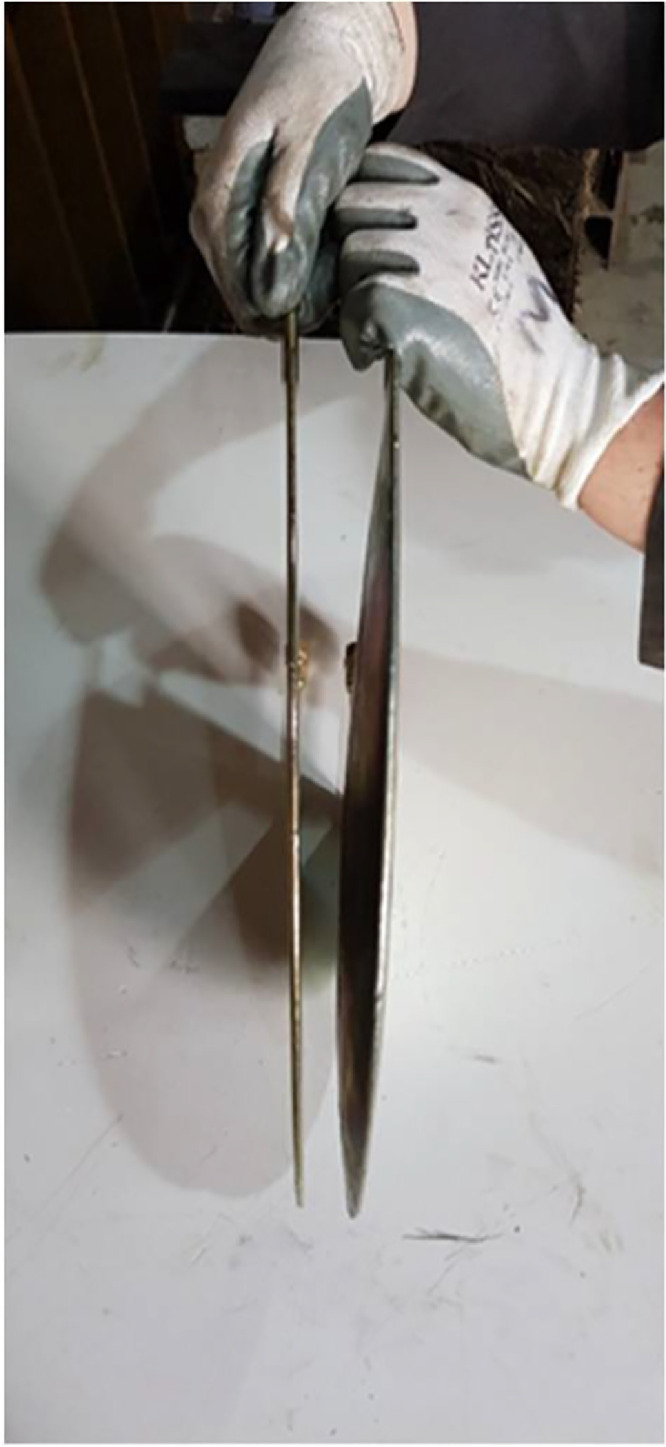
Flat jacks' comparison after test.

**Fig. 9:**
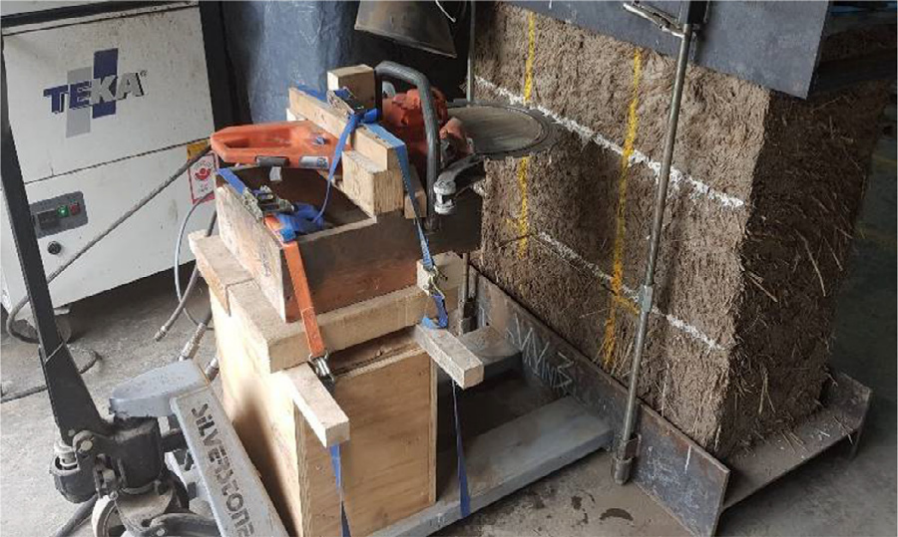
Ring saw secured in the timber frame and at the appropriate height to perform the upper cut in the cob wallettes.

The bad quality of the second cut in the first wallette prevented the successful completion of the double flat jack test. The test had to stop at a pressure value of 7.0 bars as it seemed that the flat jack was deforming excessively without actually transferring adequately the pressure to the cob wallette. It was estimated that the increment of the pressure was mainly caused by the resistance of the stiffness of the flat jack placed in the upper slot as it had overpassed by far its elastic limit. It also seemed that the flat jack placed in the lower slot started to deform beyond its elastic range as when the test was stopped and the pressure was removed from the system it was not possible to take it out from the slot. On the other hand, the uneven cut thickness allowed us to easily remove the flat jack from the upper slot and it was easy to appreciate its permanent deformation. Fig. 8 shows a comparison between the flat jack used for the single flat jack test and the flat jack introduce in the upper slot for the double flat jack test. The flat jack in the left practically recovered its original shape after the test was finished and the pressure was removed whereas that the flat jack inserted in the thick slot kept much of its deformation showing an inflated shape after removal from the slot. The cut in the rest of the wallettes was performed by simply displacing the pallets jack bellow the bottom steel pallet. The new cutting system can be seen in Fig. 6(b) for the single flat jack test and in Fig. 9 for the double flat jack test.

**Fig. 10:**
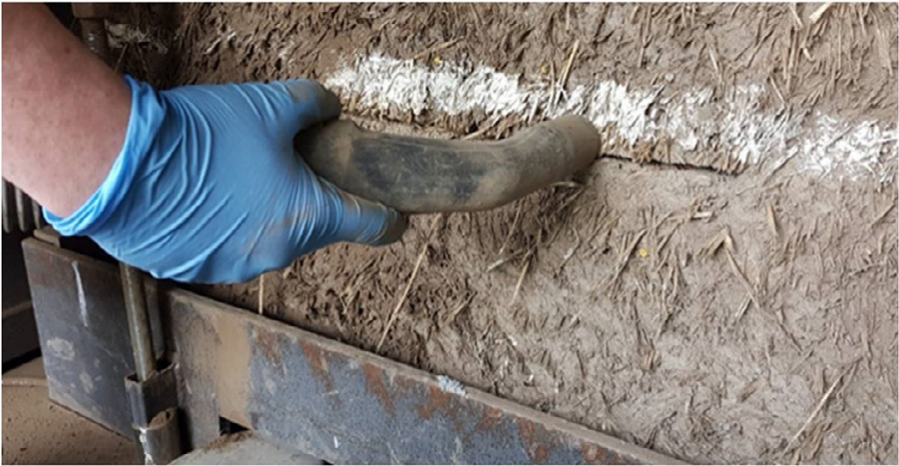
Cleaning of the slot.

It is worth mentioning that the typical onsite conditions would not allow to slide the pallets jack bellow a case study cob wall as it was the case during the experimental campaign. To deal with that situation, it would be necessary to fix the frame at the tip of the pallets jack for the ring saw's disk to protrude at the front. Thus, the cutting process could be then be performed in-situ under the same safety and precision standards as the ones reported in this paper.

### Slot cleaning

An industrial vacuum was used to clean the slot created with the ring saw before measuring the depth of the cut and insert the flatjack into it (see Fig. 43).

### Cut depth measurement

**Fig. 11:**
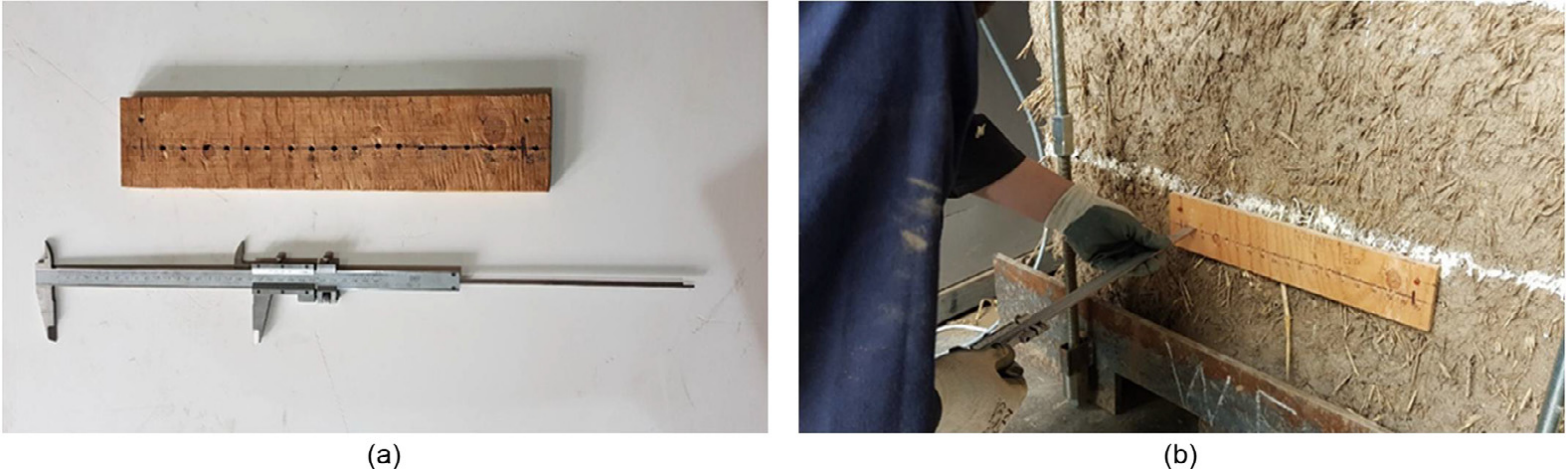
(a) Timber template and “Vernier” calipers used to measure the depth of the cut and (b) measurement of one of the cut's depth.

To measure the depth of the cut a template was fabricated with a piece of timber. The timber template was nailed to the cob wallette and as specified by the standards [2], [3], [4], [5], [6], [7] the depth of the cut was measured at every 2 cm, thus obtaining an approximate profile of the cut. The long “Vernier” calipers were used to measure the depth of the cut. The thickness of the piece of timber was of 12.3 mm and the values measured with the calipers were corrected by subtracting this value from the measurements. The template and the caliper are shown in Fig. 11(a) whereas that Fig. 11(b) shows one of the cut's depth being measured.

### Hydraulic system connection and insertion of flat jack into the slot

**Fig. 12:**
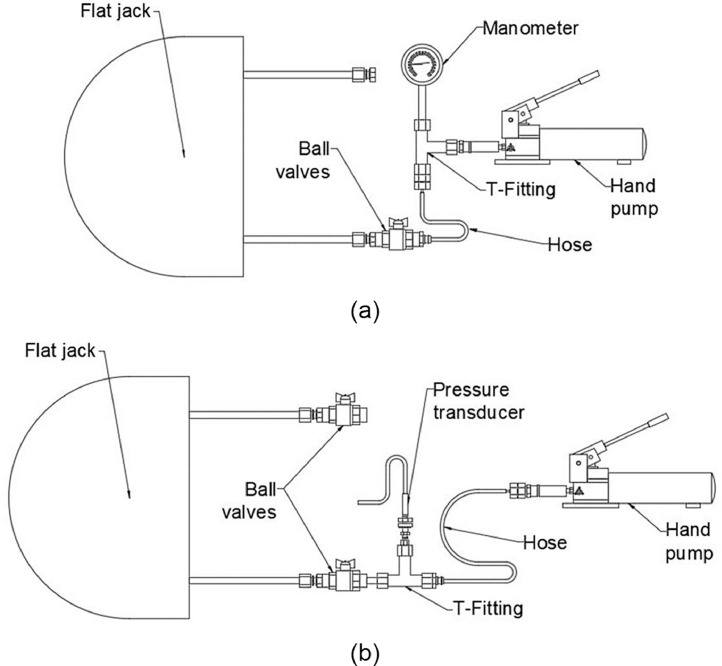
(a) Initial system connection setup proposed by flat jacks' manufacturer and (b) modified setup used in this experimental campaign.

The flat jack's manufacturer setup for the single flat jack test is shown in Fig. 12(a). This set up was slightly modified to better suit the purpose of the test. The manometer was substituted with a pressure transducer model Wykeham Farrance 28-WF6301 with a pressure range capacity of 0–2000 kPa. The pressure transducer was located just next to the ball valve connected to the inlet of the flat jack instead of directly after the pump to try to avoid peak pressure values typically appearing while manually pumping the oil. Furthermore, a second ball valve was connected to the outlet of the flat jack to improve the purging of the system and reduce the amount of trapped air within it. The final setup used is shown in Fig. 12(b).

**Fig. 13:**
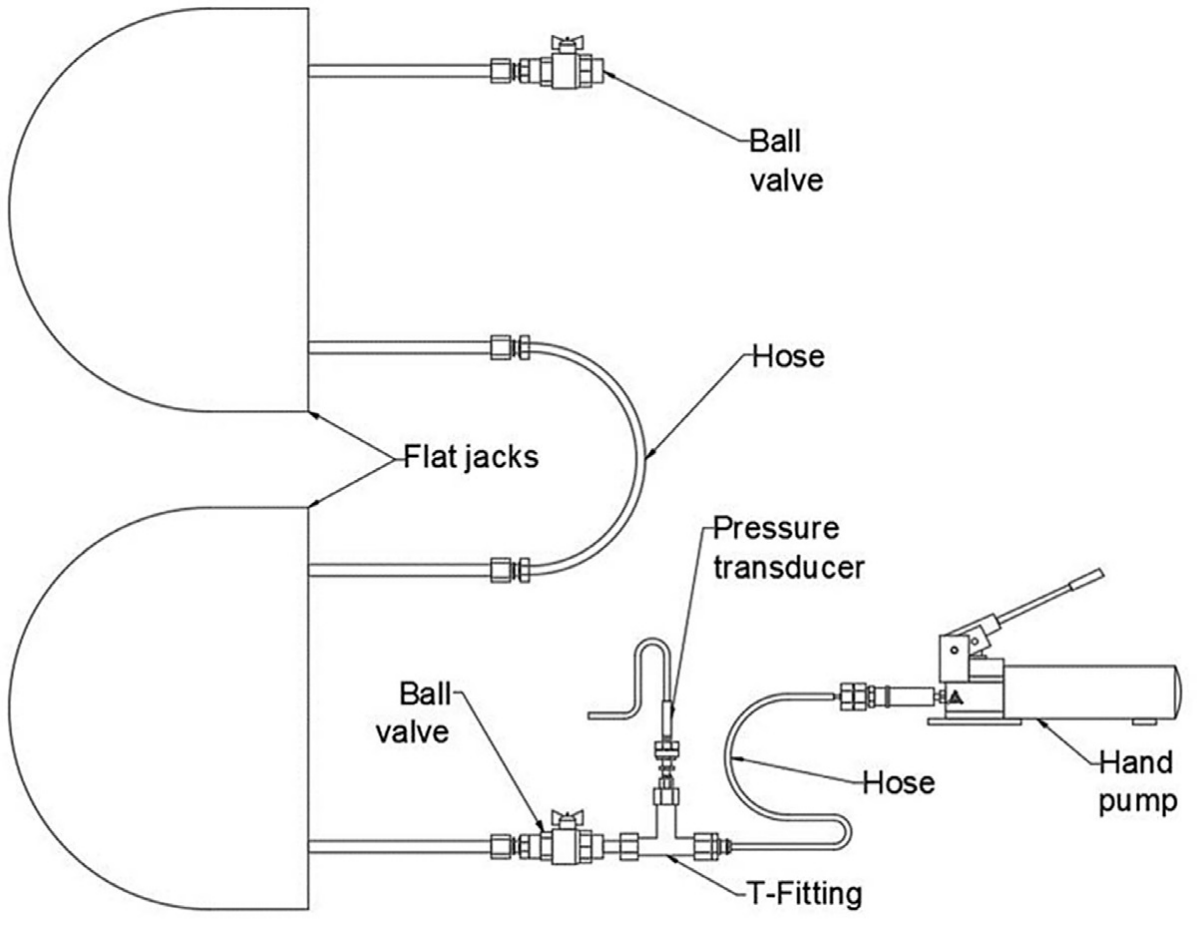
Double flat jack test system connection setup.

The hydraulic system used for the single flat jack test was slightly modified and extended by removing the ball valve at the outlet of the first flat jack and connecting a second flat jack with a hose. The ball valve was then connected at the outlet of the second flat jack. The setup for the double flat jack tests is presented in Fig. 13.

For the first cob wallette three new flat jacks were used. On the other hand, for the rest of the wallettes, the flat jack used for the single flat jack test was reused for the double flat jack test. It was decided to reuse the flat jack because after finishing the single flat jack test they had recovered their original shape and it was easy to remove them from the slot.

**Fig. 14:**
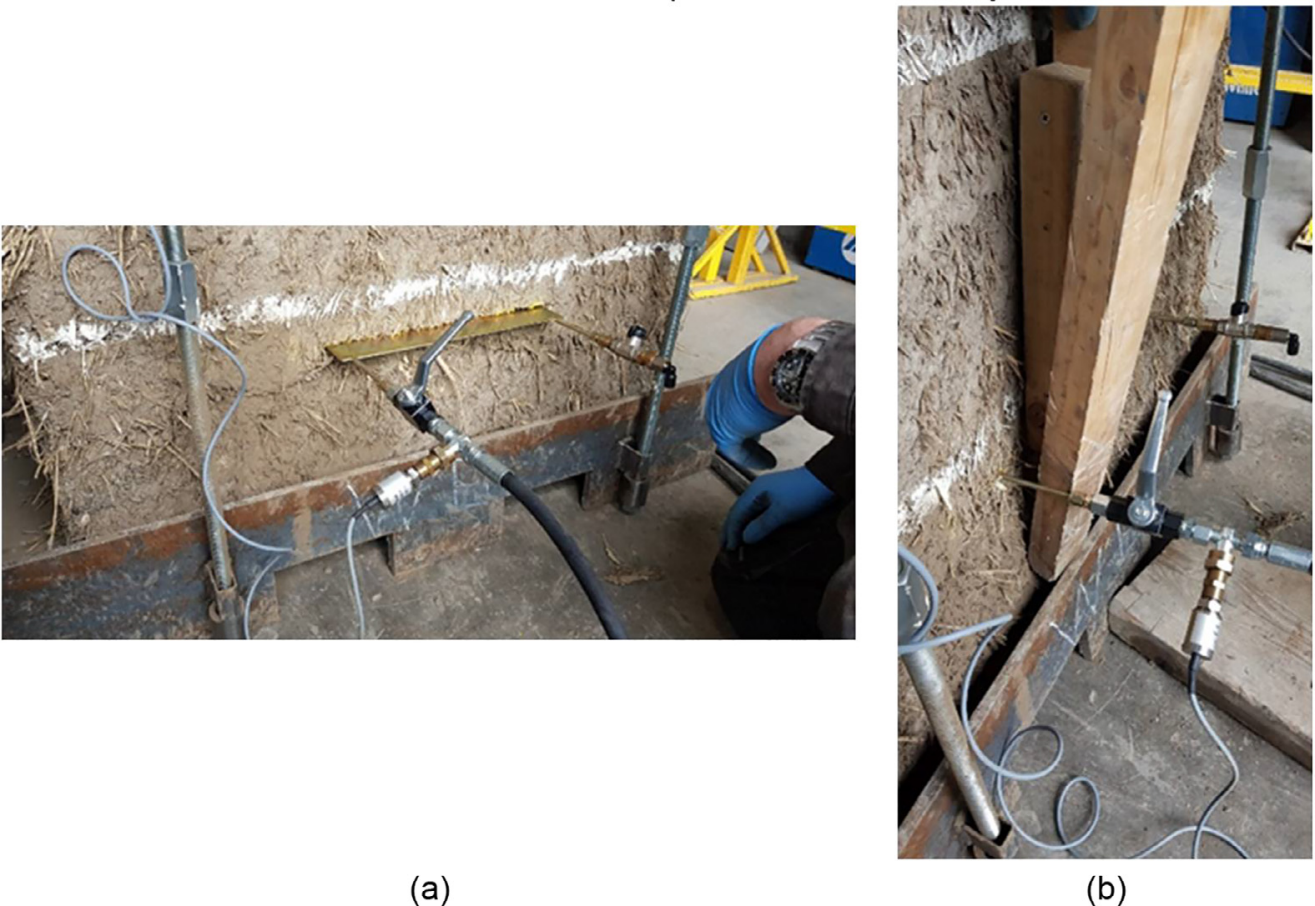
Insertion of the flat jack into the slot.

The flat jack, previously connected to the hydraulic system, was introduced into the slot. The cuts’ quality was very high and the flat jack occupied the entire thickness of the slot. In fact, the flat jack fit so tight into the slot that a few centimeters were left outside the wallettes face as shown in Fig. 14(a). From this point, the flat jack had to be gently pushed all the way through the slot by using a piece of timber as wedge (see Fig. 14(b)). The flat jack fit very tightly into the slot (except for the second slot of the first wallette) and there was no need to use steel shims to fill the extra space between the flat jack and the material.

### Hydraulic system purging, seating pressure and depressurization

**Fig. 15:**
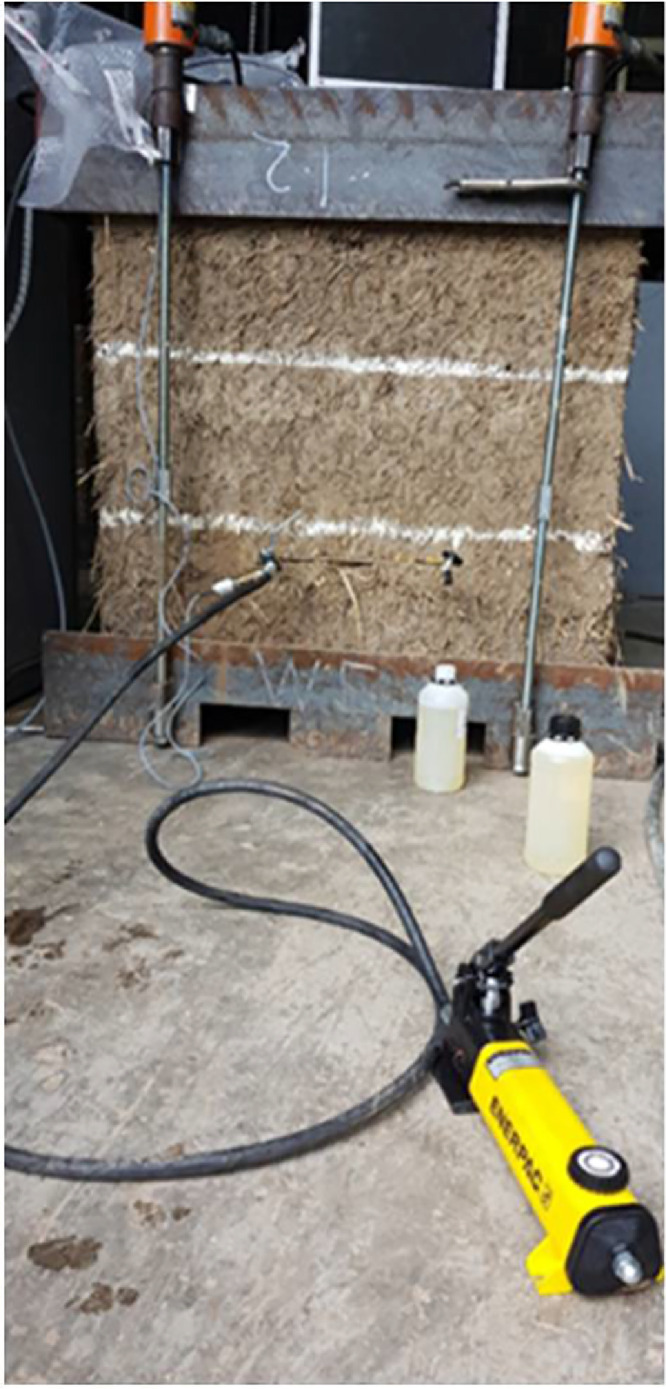
System purging.

The system was then purged, and the extra oil pumped into the system recovered in a bottle to be re-used in future tests (see Fig. 15). The ball valve installed at the end of the system enabled the closure of the system just after stop pumping in order to avoid the inclusion of air back into the hydraulic system.

After some rough calculations it was estimated that the level of stress at the location where the cut was made would be of approximately 0.14 MPa. The standards advice to apply 50% of the estimated pressure to allow the flat jack to seat into the slot. Nevertheless, a pressure of only 0.7 bars didn't seem to have any significant effect on the device. Thus, it was decided to apply 1.0 bar of pressure to allow the flat jack to seat correctly into the slot. This was also done taking into account that the standard recognizes that the single flat jack test will overestimate the level of stresses. Therefore, once the ball valve at the end of the system was closed oil was pumped to increase the pressure in the flat jack until reaching 1.0 bar. At this point the pressure was fully released.

**Fig. 16:**
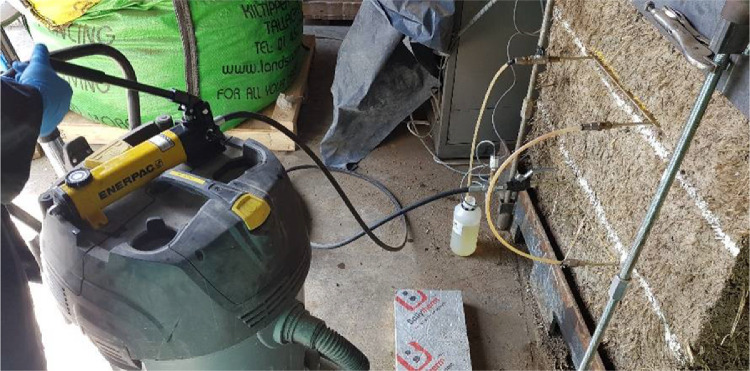
Purging of the system with both flat jacks connected.

Similarly, the system was purged by pumping oil with the hand pump for the double flat jack test. The ball valve at the end of the system was kept open and the extra oil was recovered in a bottle for further use (see Fig. 16). When no air bubbles were detected in the hoses or coming out at the end of the system the ball valve was manually closed thus preventing the inclusion of air.

Rough calculations taking into account the compressive strength determined with the cob cylinders [8] showed that a pressure of about 8.0 bars would be necessary to cause the failure in the specimen. Therefore it was decided to apply a seating pressure of 4.0 bars in the first wallette. It was difficult to achieve this value as the flat jack inserted in the top slot, which was relatively thick in comparison with the thickness of the flat jack, deformed significantly without encountering any resistance from the material. Thus, a huge amount of oil (almost 3 l) had to be pumped. A seating pressure of only 2 bars was applied to the rest of the wallettes.

Once the seating pressure was reached it was subsequently removed from the system and we proceeded to measure the initial distance between control points.

### Pressure increments and depressurization

Even though the standards [2,3] specify a pressure increment of about 0.5 bars increments of 0.25 bars were applied to the first wallette instead. This was decided based on the rough calculations performed to estimate the level of stress in the wallette. If increments of 0.5 bars were applied and the initial distance between control points turned out to be recovered at 1.5–2.0 bars the plots will not show a clear trend with only 3 or 4 points recorded. Nevertheless, after successfully performing the test in the first wallette and see that the initial distance between control points was recovered at roughly 3.5 bars it was decided to adopt pressure increments of 0.50 bars for the rest of the wallettes. Finally, after having recovered the initial distance between control points, the system was depressurized and the flat jack was easily removed from the wallette.

Pressure increments of roughly 0.5 bars were applied for the double flat jack test for the first cob wallette and at every pressure increment the distance between control points was recorded both for the vertical and horizontal control points. As it was complicated to maintain a constant pressure in the system (the pressure tended to drop as time passed while taking measurements of the distances between control points) it was decided to apply increments of 1.0 bars for the rest of the wallettes. This allowed to capture better a mean pressure value for every increment and to increase the speed of the test.

Even though the stress-strain relationship was monitored at every pressure increment the tests were not stopped when the ratio noticeably decreased as recommended by both standards [6,7]. The pressure was increased until the opening of a crack was clearly detected at the wallette's face (as the failure mode was also of interest for the authors). It was possible to reach the damage of the specimen as it did not have any historical value. Nevertheless, when the test is applied in-situ in a cob wall part of an architectural heritage building, the recommendation of the standards should be respected.

After a clear crack opening was detected in the walletes’ face the test was stopped and the system depressurized.

It is worth noting though that the test was performed relatively faster in the last wallettes in comparison to the first ones. A progressive reduction, from almost three hours to only an hour and a half, was achieved thanks to the use of the timber frame to perform the cut and to the improved level of practice acquired during the experimental campaign. Despite this reduction in the execution time of the test no time-related variations were observed in the estimation of the mechanical parameters of the cob wallettes.

## Declaration of Competing Interest

None.
